# Decreased Subcortical and Increased Cortical Degree Centrality in a Nonclinical College Student Sample with Subclinical Depressive Symptoms: A Resting-State fMRI Study

**DOI:** 10.3389/fnhum.2016.00617

**Published:** 2016-12-05

**Authors:** Cuihua Gao, Liu Wenhua, Yanli Liu, Xiuhang Ruan, Xin Chen, Lingling Liu, Shaode Yu, Raymond C. K. Chan, Xinhua Wei, Xinqing Jiang

**Affiliations:** ^1^Guangzhou First People’s Hospital, Guangzhou Medical UniversityGuangzhou, China; ^2^Faculty of Health Management, Guangzhou Medical UniversityGuangzhou, China; ^3^Shenzhen Institutes of Advanced Technology, Chinese Academy of ScienceShenzhen, China; ^4^CAS Key Laboratory of Mental Health, Institute of Psychology, Chinese Academy of SciencesBeijing, China

**Keywords:** subclinical depression, young adults, magnetic resonance imaging, resting-state functional connectivity, degree centrality

## Abstract

Abnormal functional connectivity (FC) at rest has been identified in clinical depressive disorder. However, very few studies have been conducted to understand the underlying neural substrates of subclinical depression. The newly proposed centrality analysis approach has been increasingly used to explore the large-scale brain network of mental diseases. This study aimed to identify the degree centrality (DC) alteration of the brain network in subclinical depressive subjects. Thirty-seven candidates with subclinical depression and 34 well-matched healthy controls (HCs) were recruited from the same sample of college students. All subjects underwent a resting-state fMRI (rs-fMRI) scan to assess the DC of the whole brain. Compared with controls, subclinical depressive subjects displayed decreased DC in the right parahippocampal gyrus (PHG), left PHG/amygdala, and left caudate and elevated DC in the right posterior parietal lobule (PPL), left inferior frontal gyrus (IFG) and left middle frontal gyrus (MFG). In addition, by using receiver operating characteristic (ROC) analysis, we determined that the DC values in the regions with altered FC between the two groups can be used to differentiate subclinical depressive subjects from HCs. We suggest that decreased DC in subcortical and increased DC in cortical regions might be the neural substrates of subclinical depression.

## Introduction

Subclinical depression, defined as relevant depressive symptoms without meeting the full criteria of a clinical depressive disorder, has been identified as a health problem among college students worldwide (Mikolajczyk et al., 2008a). A body of evidence indicates that individuals with subclinical depression have an increased risk of later major depression and other adverse outcomes (Flett et al., 1997; Fergusson et al., 2005). Despite a high prevalence among college students (Mikolajczyk et al., 2008a,b), the underlying neural substrates of subclinical depression remain poorly understood.

A recent review indicated altered functional activations in the extended medial prefrontal network regions in youth major depression disorder (MDD), including the anterior cingulate cortex (ACC), ventromedial and orbitofrontal cortices and subcortical areas such as the amygdala and striatum (Kerestes et al., 2013). Moreover, evidence indicates abnormal functional connectivities (FCs) between brain regions rather than within brain regions in MDD. Specifically, dysregulation of frontal-subcortical connectivity (e.g., frontolimbic circuits) involved in the neural circuit mediating emotion perception and mood regulation (Cao et al., 2012) has been proposed to account for both affective and cognitive symptoms in MDD subjects (Mayberg, 2003; Cao et al., 2012). Although alterations among brain regions have been reported in MDD patients, whether the same alterations are present in subclinical or subthreshold depression remains largely unknown. A few brain imaging studies on the neurobiology of subthreshold depression have been carried out recently. For example, a voxel-based morphometry (VBM) study revealed decreased gray matter volume in the right parahippocampal gyrus (PHG) in elderly individuals with subthreshold depression (Zhou et al., 2016); in addition, compared with controls, smaller gray matter volume in the frontolimbic circuits including the prefrontal, ACC, caudates and cingulum was found in adolescents with subthreshold depression (Vulser et al., 2015). These studies may partially indicate neural substrates related to structural alterations of subthreshold depression; however, the changes in FC between these brain regions with structural alterations remain unknown.

In recent years, resting-state fMRI (rs-fMRI) has been extensively used to reveal alterations of brain function (Rosazza and Minati, 2011; Lee et al., 2012). Among the methods for analysis of rs-fMRI data, FC approaches describing the relationships between distinct brain areas based on the correlations between blood oxygenation level-dependent time series are increasing used to explore the pathophysiology of neuropsychiatric disorders (Ma et al., 2010; Hacker et al., 2012). The seed-based FC and independent component analysis (ICA) approaches are the most commonly used FC methods and have been proven useful in examining connectivity patterns for distinct brain regions or specific components of interest (Lee et al., 2012). In seed-based FC analysis, a seed region is selected *a priori*, and the subsequent FC map is extracted from the temporal correlations between the regional of interest (ROI) and all other voxels in the brain or other distributed ROIs (Margulies et al., 2010). ICA is a mathematical method that maximizes statistical independence among its components. Compared with a seed-based approach, the ICA approach proceeds without *a priori* selection (Lee et al., 2012). However, it can be difficult to determine whether a component represents physiological noise or a brain network (Rosazza et al., 2012). Recent studies are increasingly accepting the view that the brain consists of complex large-scale networks characterized by interregional interactions (de Pasquale et al., 2013; Zahr, 2013). The newly proposed degree centrality (DC) approach is drawing intense attention because it is the most reliable metric among several large-scale network metrics (Li et al., 2016). This graph-based method measures functional relationships between a region and the rest of the brain within the entire connectivity matrix of the brain (connectome) at the voxel level (Zuo et al., 2012). Therefore, DC is a better network metric than other measurements because it counts the number of direct connections for a given voxel in a network and reflects its FC within the brain network without requiring *a priori* selection. The DC approach has recently been used in mental disorders including MDD (Zhang et al., 2016), hepatic encephalopathy (Qi et al., 2015), schizophrenia (Zhuo et al., 2014), multiple sclerosis (Zhuang et al., 2015), and autism and attention-deficit (Di Martino et al., 2013). However, no study so far has investigated DC alterations in subclinical depression. As there have been very few studies focused on subclinical depression in young adults and the brain alterations of subclinical depression are largely unknown (Wei et al., 2014, 2015), the DC approach without requiring *a priori* selection is a promising strategy to uncover the neural basis of subclinical depression.

Therefore, the objective of the present study was to explore centrality analysis through a graph-theoretical approach in subjects with subclinical depression using rs-fMRI data. Specifically, we sought to identify the alterations of DC in subclinical depressive subjects compared with well-matched healthy controls (HCs) and explore how these measures of DC relate to the presence of depressive scores. Furthermore, as part of an effort to identify neuroimaging markers of subclinical depression, we sought to determine whether the DC values can be used to differentiate the subclinical depressive subjects from HCs. We hypothesized that subjects with subclinical depression would have disrupted functional brain topological organization and the values of DC could be used as a biomarker to identify subclinical depression.

## Materials and Methods

### Participants

The participants were recruited as volunteers who had undergone a private health screening at Guangzhou Medical University between 2012 and 2014. Depressive symptoms were measured using the Beck Depression Inventory (BDI)-II scale, which is a widely used self-report inventory for assessing the severity of depressive symptoms. This revision of the BDI is consistent with DSM-IV major depressive episode diagnostic criteria (Cukrowicz et al., 2011) and has good reliability and validity in both healthy and depressed samples (Ho et al., 2014). The BDI-II consists of 21 items that are rated on a 4-point scale and is scored by summing the highest ratings for each of the 21 symptoms. Sum scores range from 0 to 63. Scores between 0 and 13 indicate minimal, between 14 and 19 mild, between 20 and 28 moderate and between 29 and 63 severe depression (Dolle et al., 2012).

In this study, 37 subjects (10 male, 27 female) who scored more than 13 on the BDI-II were enrolled in the subclinical depressive group. During the same period, a total of 34 control subjects (male 11, female 23) who scored less than 4 on the BDI-II were randomly selected for comparison after matching by age, sex and education to the subclinical depressive subjects. None of the subjects in either group fulfilled the criteria for MDD in the DSM-IV. Other inclusion criteria were as follows: age range from 19 to 25 years, right-handedness, no visualized lesion on MRI, no neurological illness and no alcohol or drug dependence.

This research was performed in accordance with the ethical guidelines of the Declaration of Helsinki (version 2002) and was approved by the Medical Ethics Committee of Guangzhou First People’s Hospital of Guangzhou Medical University. All participants provided written informed consent.

### Imaging Data Acquisition

MRI data were obtained on a 3-Tesla MRI scanner (Siemens, Erlangen, Germany) using an 8-channel brain phased-array coil. Foam pads were used to minimize subject head motion, and headphones were used to reduce scanner noise. Consistent with our previous study (Wei et al., 2015), rs-fMRI scans were obtained with gradient-echo echo planar imaging (TR = 2500 ms, TE = 21 ms, FA = 90°, FOV = 200 mm × 200 mm, matrix = 64 × 64, voxel size = 3.5 mm ×3.1 mm ×3.1 mm, 42 slices, no gap) covering the entire brain. After the functional MR scan, a high-resolution T1-weighted structural image was acquired with the following parameters: TR = 2530 ms, TE = 2.34 ms, FA = 7°, FOV = 256 mm × 224 mm, 1.0 mm thickness, no gap, 1 acquisition.

### Preprocessing of Functional Imaging Data

Functional MRI data preprocessing was performed using the Data Processing Assistant for rs-fMRI advanced edition (DPARSF; Chao-Gan and Yu-Feng, 2010)^1^, which works with SPM8^2^ implemented in the MATLAB (The Math Works, Inc., Natick, MA, USA) platform. Consistent with our previous work (Wei et al., 2015), before the preprocessing, the first 10 functional volume images of each subject’s dataset were discarded due to magnetization effects. Then, the remaining rs-fMRI data were corrected for slice timing and realigned for motion correction. The standard Montreal Neurological Institute (MNI) template provided by SPM8 was used for spatial normalization with a resampling voxel size of 3 mm × 3 mm × 3 mm. No subjects had head motion exceeding 3 mm of movement or 3° rotation in any direction. The covariates including the white matter signal, cerebrospinal fluid signal and Friston 24 motion parameters, were regressed out from the time series of every voxel. The rs-fMRI dataset was then filtered using a typical temporal bandpass (0.01–0.08 Hz) to reduce the low-frequency drift and high-frequency respiratory and cardiac noise. Given the controversy of removing the global signal in the preprocessed rs-fMRI data (Murphy et al., 2009), we did not regress the global signal out in the present study.

### DC Calculation

Weighted DC measures were calculated using the “REST-DC” toolkit in the REST V1.8 package^3^ (Zuo et al., 2012), as previously described (Di Martino et al., 2013; Liu et al., 2015; Li et al., 2016). Briefly, to obtain each participant’s graph, Pearson correlation coefficients were computed between the time series of all pairs of brain voxels. Each voxel represented a node in the graph, and each significant functional connection (i.e., Pearson correlation) between any pair of voxels was an edge. As a result, we obtained an n × n matrix of Pearson correlation coefficients between any pair of voxels to construct the whole-brain FC matrix for each participant. Then, individual correlation matrices were transformed into a *Z*-score matrix using Fisher’s r-to-z transformation to improve normality (Takeuchi et al., 2015). The weighted DC strength of a voxel as the sum of the connections (*Z*-values) between a given brain voxel and all other voxels was then computed. As previously described (Li et al., 2016), to eliminate possible spurious connectivity, we used thresholded the Pearson correlation coefficient at *r* > 0.25 by thresholding each correlation at *P* ≤ 0.001. Furthermore, standardized weighted DC maps were acquired by subtracting the mean value, and then dividing by the standard deviation within the whole gray matter mask (Zuo et al., 2012; Takeuchi et al., 2015). Finally, the resulting DC maps were spatially smoothed with a 6-mm full width at half maximum (FWHM) Gaussian kernel.

Because the choice of this threshold was arbitrary, DC maps were also calculated based on two different correlation thresholds (i.e., 0.2 and 0.3) to examine whether our primary results were dependent on the chosen threshold. In the Supplementary Material section, we provide detailed DC maps with the additional correlation thresholds.

### Statistical Analysis

Statistical analysis was performed using SPSS version 16.0 (SPSS Inc., Chicago, IL, USA). Independent two-sample *t*-tests and the chi-squared test were used to assess the differences in demographic data and BDI scores between subclinical depressive subjects and HCs. A *P*-value <0.05 was deemed significant.

DC analyses were carried out using the REST V1.8 package^4^ (Song et al., 2011). First, to explore the within-group DC patterns (to evaluate whether DC differed from the value of one), a one-sample *t*-test (*P* < 0.05, AlphaSim corrected) was performed on the individual normalized DC maps for each group (subclinical depression and HC). Then, to explore the differences in DC between the subclinical depressive subjects and controls, a two-sample *t*-test was performed in a voxel-by-voxel manner, and the age, gender and gray matter volume of each subject were taken as covariates to avoid any undetected effects (Jenkinson et al., 2002). All results were presented at the statistical threshold of *P* < 0.001 using AlphaSim correction, as determined by Monte Carlo simulations^5^. Using this program, clusters that were greater than 13 voxels were applied to the resulting statistical map at a corrected significance level of *P* < 0.001.

To investigate the correlation between depressive scores and DC values in regions with significant group differences in depressive subjects, the average DC values of all voxels within the ROIs revealed by DC analysis were extracted separately using the REST package. Then, a bivariate correlation using SPSS 16.0 was introduced to explore the correlation between the DC values and BDI scores, and the significance level was set at *P* < 0.05 (two-tailed).

Moreover, to explore whether DC measures can be used to distinguish subclinical depressive subjects from HCs, receiver operating characteristic (ROC) curve analysis was used to summarize the area under the curve (AUC) and sensitivity/specificity characteristics of regions with alterations of DC measures between two groups, and the optimal cut-off DC values were determined.

## Results

### Clinical Data and Depressive Tests

No differences in gender, age or education level were found between subclinical depressive subjects and HCs (*P* > 0.05). As expected, subclinical depressive subjects had higher BDI scores compared with HCs (*P* < 0.05; Table 1).

**Table 1:** **Demographics and depressive scores of the participants**.

Characteristic	Depressive subjects (*n* = 37)	Healthy controls (*n* = 34)	*P* value
Sex (male/female)	14/23	15/19	0.593^a^
Age (years)	19.81 ± 1.56	19.29 ± 1.001	0.105^b^
Education (years)	13.16 ± 0.688	13.03 ± 0.627	0.337^b^
BDI score	23.84 ± 7.89	1.18 ± 1.70	0.000^a^

^a^*The *P* value for gender distribution in the two groups was obtained by the Chi-square test. ^b^The *P* values for differences in age, years of education and BDI score between the two groups were obtained by the two-sample *t* test. Values are expressed as the mean ± SD. BDI, Beck Depression Inventory*.

### DC Analysis

**Figure 1:**
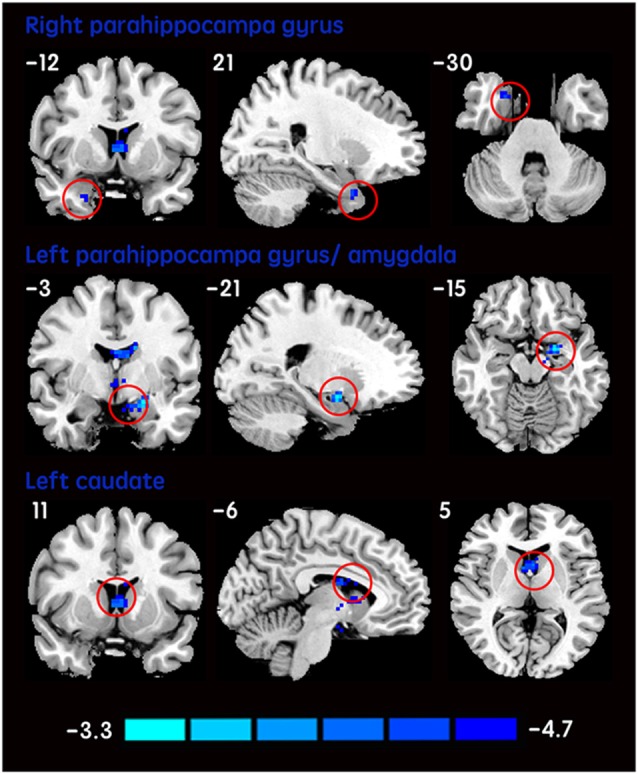
**DCs are decreased in subclinical depressive subjects.** Brain regions exhibiting decreased (red) DC values in subclinical depressive subjects compared with healthy controls (HCs; two-sample *t*-tests, with a *P* < 0.001 threshold, corrected) are shown on coronal, sagittal and axial views with the MNI location. The color bar indicates the *T* score. DC, degree centrality; MNI, Montreal Neurological Institute.

**Figure 2:**
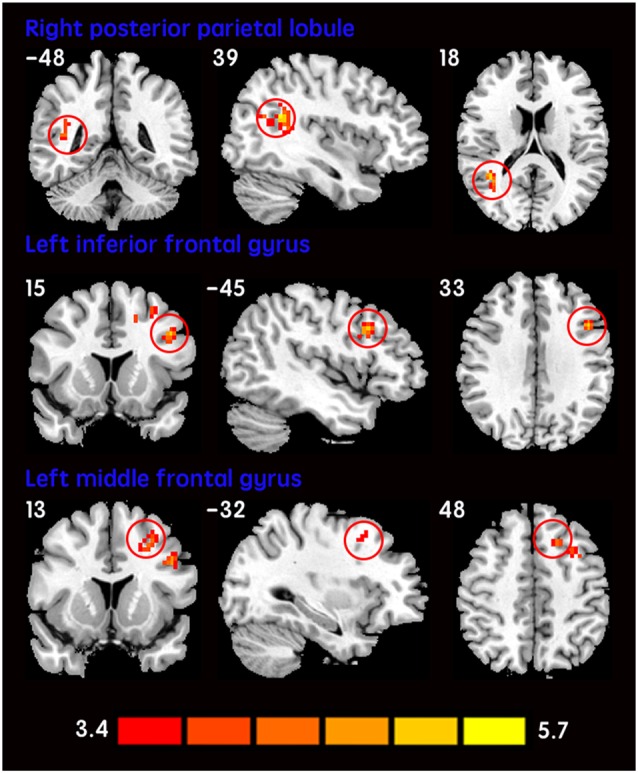
**Increased DCs in subclinical depressive subjects.** The brain regions (red) demonstrating increased DCs in subclinical depressive subjects compared with HCs are presented on coronal, sagittal and axial views with the MNI location. The color bar indicates the *T* score (two-sample *t*-tests, with a *P* < 0.001 threshold, corrected). DC, degree centrality; MNI, Montreal Neurological Institute.

**Figure 3:**
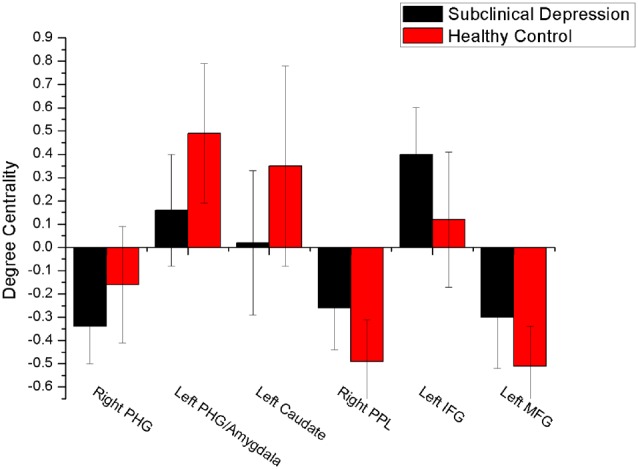
**Comparison of DC values between subclinical depression group and controls.** There were significant difference in DC values between the depressive subjects and controls in six brain regions. PHG, parahippocampal gyrus; PPL, posterior parietal lobule; vPCC, ventral posterior cingulate cortex.

The mean DC maps for both groups (subclinical depression and HC) are shown in the Supplementary Material section (Figure S1). Compared with HCs, subclinical depression subjects exhibited decreased DCs in the right PHG, left PHG/amygdala and left caudate (Figure 1; Table 2) and increased DCs in the right posterior parietal lobule (PPL), left inferior frontal gyrus (IFG) and left middle frontal gyrus (MFG; Figure 2; Table 2). The DC values in these brain regions in both groups are compared in Figure 3. In addition, the results for the between-group differences in DC spatial distribution maps were highly similar and were not dependent on the different correlation thresholds (Figure S2).

**Table 2:** **Difference of degree centrality in subclinical depressive subjects and control subjects**.

Brain region	BA	Voxels size	Peak MNI coordinates (mm)			*T* value
			*X*	*Y*	*Z*	
**Subclinical depressive subjects < Healthy controls**
Right parahippocampa gyrus	36	18	21	−12	−30	−3.8951
Left parahippocampa gyrus/amygdala	28	40	−21	−3	−15	−5.3539
Left caudate	25	72	−6	11	5	−4.9123
**Subclinical depressive subjects > Healthy controls**
Right posterior parietal lobule	39	84	39	−51	21	6.0549
Left inferior frontal gyrus	44	32	−45	15	33	5.5371
Left middle frontal gyrus	9	46	−32	13	48	5.2102

*BA, Brodman’s area; MNI, Montreal Neurological Institute. *P* < 0.001, corrected for multiple comparisons*.

Given there is a controversy regarding whether the global signal should be regressed out in the rs-fMRI analysis. We checked how the results of this study were when mean time course of whole brain was regressed out in individual analyses. There were similar tendencies for DC when the mean time course of whole brain was regressed out (Figure S3).

### Correlation Between DC Values and BDI Scores

No significant correlation was observed between DC values and BDI scores in the brain regions with altered DC in subclinical depressive subjects.

### ROC of DC Values Analysis

**Figure 4:**
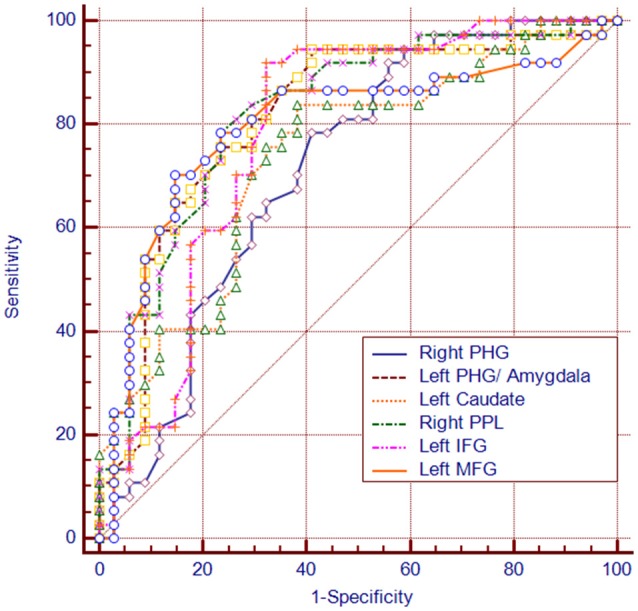
**The diagram of the receiver operating characteristic (ROC) curve for DC values in brain regions with altered DC.** PHG, parahippocampal gyrus; PPL, posterior parietal lobule; vPCC, ventral posterior cingulate cortex.

Among the regions that exhibited altered DCs in both groups, the AUCs ranged from 0.774 to 0.865 in the right PHG, left PHG/amygdala, left caudate, right PPL, left IFG and left MFG (Figure 4; Table 3).

**Table 3:** **The ROC analysis of degree centrality in subclinical depressive individuals and control subjects**.

Brain regions	AUC	Sensitivity	Specificity	Cut-off
Right parahippocampa gyrus	0.712	78.4%	58.8%	−0.27
Left parahippocampa gyrus/amygdala	0.815	94.6%	58.8%	0.47
Right caudate	0.736	83.8%	61.8%	0.23
Right posterior parietal lobule	0.821	78.4%	76.5%	−0.39
Left inferior frontal gyrus	0.783	91.9%	67.7%	0.17
Left middle frontal gyrus	0.795	70.3%	85.3%	−0.41

*AUC, area under curve*.

## Discussion

This study compared DCs in subjects with subclinical depressive symptoms and those in a group of well-matched HCs. The present study identified significantly decreased DCs in subcortical regions including the right PHG, left PHG/amygdala and left caudate and increased DCs in several cortical regions, such as the right PPL, left IFG and left MFG in subclinical depressive participants. By using ROC analysis, we observed that the DC values in the regions with alterations of FC can be used to differentiate subclinical depressive subjects from HCs.

In the present study, we observed decreased DCs in some subcortical brain regions including the right PHG, left PHG/amygdala and left caudate in subjects with subclinical depressive symptoms. The PHG is associated with regulatory function during emotional processing and has rich connections with the amygdala (Frey et al., 2007). Both regions are reciprocally and intensively interconnected functionally and anatomically and are frequently coactivated during the performance of emotional tasks in fMRI studies of normal emotional processing (Phelps, 2004). Specifically, the PHG is involved in memory encoding and retrieval. In particular, the PHG may play a critical role in providing an interface for the interaction of emotion, cognitive evaluation and episodic autobiographical memory for helping identify the social and emotional context of an episode (Aggleton, 2012). Therefore, dysfunctional parahippocampal circuits may disrupt limbic pathways involved in affect regulation (Cao et al., 2015). The amygdala has been consistently considered to play a central role in both emotional perception and arousal (LeDoux, 2000). Abnormalities in the amygdala were found in MDD patients in structure MRI (Cullen et al., 2010; Arnold et al., 2012), rs-fMRI (Anand et al., 2009; Veer et al., 2010) and task-fMRI studies. The amygdala is supposed to be involved in facilitating emotional memory and generating an autonomic emotional response (Groenewold et al., 2013). Interestingly, consistent with previous studies in unipolar depression (Peluso et al., 2009), lateralization of the left amygdala with abnormality was observed in subclinical subjects in the present study. However, these findings are not universal (Farahbod et al., 2010). Whether the lateralization of amygdala dysfunction is a feature of depressive subjects requires more evidence. Given the role of the amygdala and PHG in modulating emotional behavior, dysfunction of neural circuits involving these regions indicates that neural network communication is impaired and involved in bias mood processing and cognition, which might underlie the pathogenesis of subclinical depression. The caudate is another brain region in which abnormality is commonly reported in depressive disorder patients (Zhang et al., 2013). As a central part of the reward circuit, in contrast to the amygdala, the caudate is more often associated with the processing of rewards (Lorenz et al., 2014) and positive emotion (Haber and Knutson, 2010). A previous structural study in non-clinical participants reported that trait anhedonia is linked to volumetric reduction in the caudate (Zhang et al., 2013). Considering the decreased DC in the left caudate and amygdala observed in the current study, we suggest that subclinical subjects might exist negativity bias toward presenting negative information and inhibiting positive information for further processing of emotion.

Increased DC values were observed mainly in the right PPL (Brodman’s area [BA 39]), left IFG (BA 44) and left MFG (BA 9) in subclinical depressive subjects. The PPL is components of the default mode network (DMN; Raichle et al., 2001; Franco et al., 2009; Dutta et al., 2014), which is most active at rest and may be related to negative rumination and self-referential processing (Bluhm et al., 2009). Specifically, the anterior medial regions of the resting-state DMN are associated with rumination, whereas the posterior medial regions are associated with overgeneral autobiographical memory, which is a risk factor for the onset and course of depression (Zhu et al., 2012; Onnink et al., 2014). Therefore, given the increased DC values observed in the right PPL, we speculate that overgeneral autobiographical memory may be a neural substrate of subclinical depression. In addition, increased DC in the left IFG (BA 44) was observed in subclinical depressive subjects in the present study. Similarly, the abnormalities of left IFG have been increasingly reported in MDD (Guo et al., 2012) and nonclinical depression (Wei et al., 2014). It was suggested that deficits in semantic labeling of negative emotions were related to increased activation in left IFG in medication-free depressed individuals (van Wingen et al., 2011). Moreover, deficits in emotion recognition appeared after damage to the frontal operculum (involving BA 44; Adolphs et al., 2002). Therefore, in addition to language-related function, the left IFG (BA 44) is implicated in emotion recognition. We suppose that the increased recruitment of the left IFG may reflect an attempt to compensate for inadequate behavior. Apart from the left IFG, we also observed increased DC in the left MFG (BA 9) in the left prefrontal cortex (PFC) areas in the subclinical depressive subjects. BA 9, a component of dorsal lateral PFC (DLPFC), was considered to play a critical role in cognitive processing (Fossati et al., 2002; Comte et al., 2016). In fact, cognitive impairment is one of the key characteristics of depressive patients (Gotlib and Joormann, 2010) and subthreshold depressive individuals (Wesselhoeft et al., 2013). The current result is consistent with previous studies reporting an increased rs-FC of the DLPFC in subclinical depressive individuals (Laeger et al., 2012). However, this is in contrast to a preponderance of findings showing decreased DLPFC activity in depressive patients (Taylor and Liberzon, 2007; Wang et al., 2008). This could be explained in part by differences in neural basis between clinical and subclinical depression. Together these findings, we suggest that an increased DC of DLPFC is necessary to achieve or maintain an optimal or “near-normal” level of cognitive performance. Together, the increased recruitment of the left IFG and MFG, may reflect an attempt to compensate for inadequate behavior in subclinical depression.

However, in the current study, we did not find significant correlations between the mean DC values in the regions showing significant differences and depressive scores in subclinical depressive subjects. The potential reasons are as follows: first, the relatively small ample size may lead to insufficient statistical power. Second, we did not divide the subclinical depressive subjects into more subgroups based on the depressive scores. As a result, a compound effect of the different degrees of depressive symptoms could not be avoided.

Importantly, the sensitivity and specificity of the DC values in the ROIs with altered DC measures were all greater than 70% in the ROC analysis, which means that the DC values in these ROIs can be used as reliable biomarkers to differentiate the individuals with subclinical depression from HCs. Nevertheless, a larger sample size is needed in future studies to support this result.

Our study has several limitations that must be acknowledged. First, the present study is cross-sectional and therefore cannot address whether or not these observations are a consequence of subclinical depression. Future longitudinal studies should be conducted to address this question. Second, given the high rates of comorbid symptoms (e.g., anxiety) in this sample of subclinical depressed adolescents, future studies are needed to investigate the specificity of these findings and how they might be influenced by comorbidity. Third, the effect of gender differences should be evaluated. Future studies are required to address how DC varies by gender in subjects with subclinical depression.

Taken together, our data provide novel insights into the neural basis of subclinical depression. We observed decreased DCs in a few subcortical regions and increased DCs in several cortical areas in subclinical depression subjects. Furthermore, we propose that DC values might be useful as imaging biomarkers for the early diagnosis of subclinical depression.

## Author Contributions

CG was involved in literature review, data collection and writing of the manuscript. XW contributed in the experimental design and revision of the manuscript. LW contributed to the data collection and analysis of neuropsychological data. LL, SY and YL contributed to the analysis of MRI data. YL, XR and XC was involved in the data collection. RCKC was involved in revision of the manuscript. XJ contributed in the experimental design.

## Conflict of Interest Statement

The authors declare that the research was conducted in the absence of any commercial or financial relationships that could be construed as a potential conflict of interest.

## Footnotes

^1^http://www.rest.restfmri.net

^2^http://www.fil.ion.ucl.ac.uk/spm/software/SPM8

^3^http://www.restfmri.net

^4^http://resting-fmri.sourceforge.net

^5^https://afni.nimh.nih.gov/pub/dist/doc/program_help/AlphaSim.html

## Supplementary Material

Figure S1 **The mean degree centrality (DC) maps of two groups:** healthy control **(A)** and subclinical depression **(B)**.

Figure S2 **Comparison of degree centrality (DC) maps based on different Pearson’s correlation coefficient threshold between subclinical depression and healthy control:** *r* > 0.2 **(A)**, *r* > 0.25 **(B)**, and* r* > 0.3 **(C)**. All the DC maps were calculated by thresholding each correlation at *P* ≤ 0.001. The brain regions with significantly increased (red) or decreased (blue) DC in the subclinical depressive subjects vs. the healthy controls are shown in the lateral, medial and dorsal views of 3D brain templates. The threshold was *P* < 0.001 (corrected).The visualization used the Brain Net Viewer software (http://www.nitrc.org/projects/bnv).

Figure S3 **The degree centrality (DC) maps calculated with removing the global signal in the preprocessed resting-state fMRI data in the subclinical depression vs. healthy control.** There were similar tendencies for DC when the mean time course of whole brain was regressed out (compare with Figure S2B).
