# Supplementary figures and images for: Decreased Subcortical and Increased Cortical Degree Centrality in a Nonclinical College Student Sample with Subclinical Depressive Symptoms: A Resting-State fMRI Study
Source: Front Hum Neurosci. 2016 Dec 5;10:617. doi: 10.3389/fnhum.2016.00617 (PMC5136555; doi:10.3389/fnhum.2016.00617)

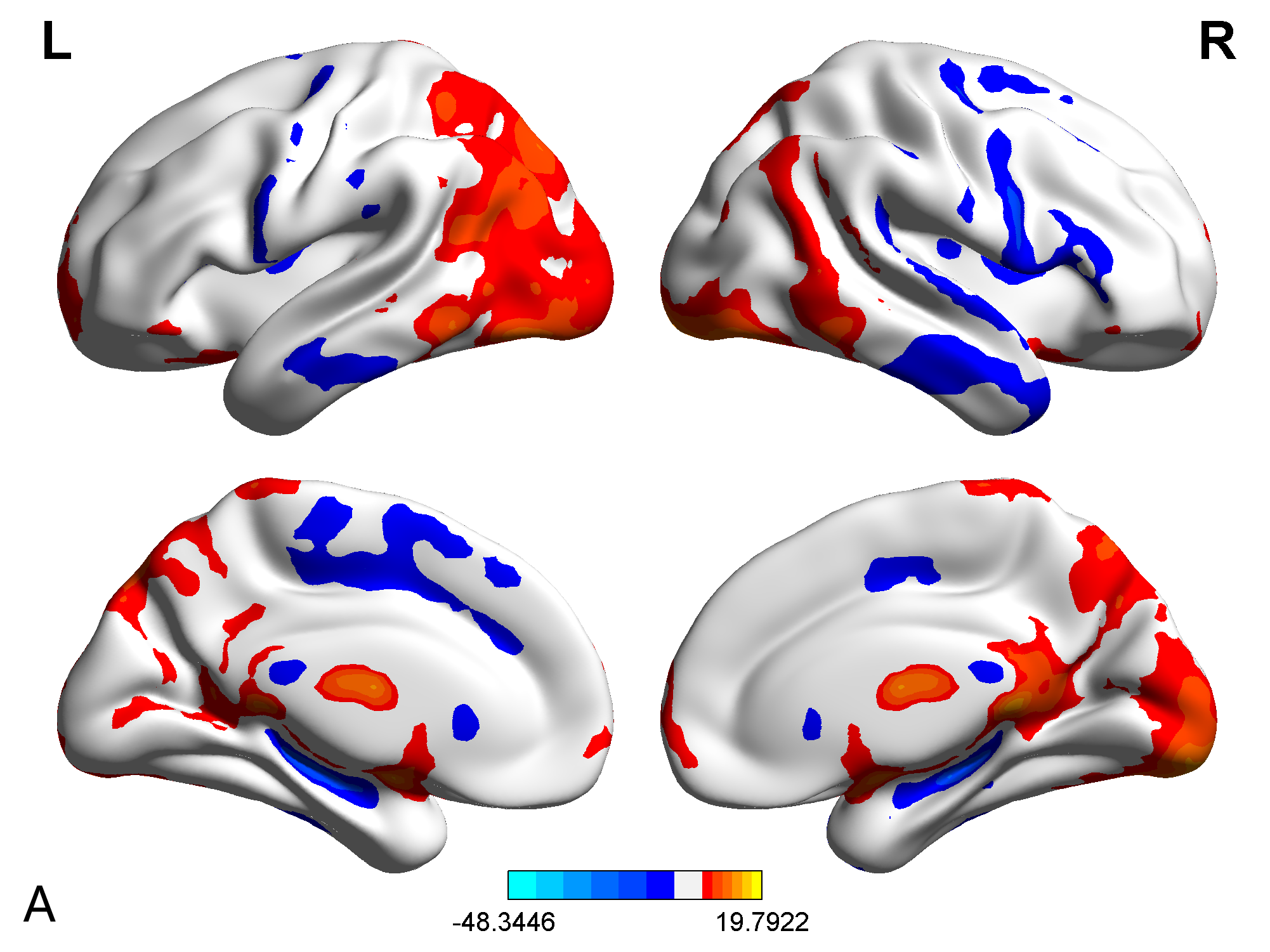

Supplement: Figure S1 — The mean degree centrality (DC) maps of two groups: healthy control (A) and subclinical depression (B). [file DataSheet_1.zip › Suppl Mat 10.3389fnhum.2016.00617/Figure S1A.tif]

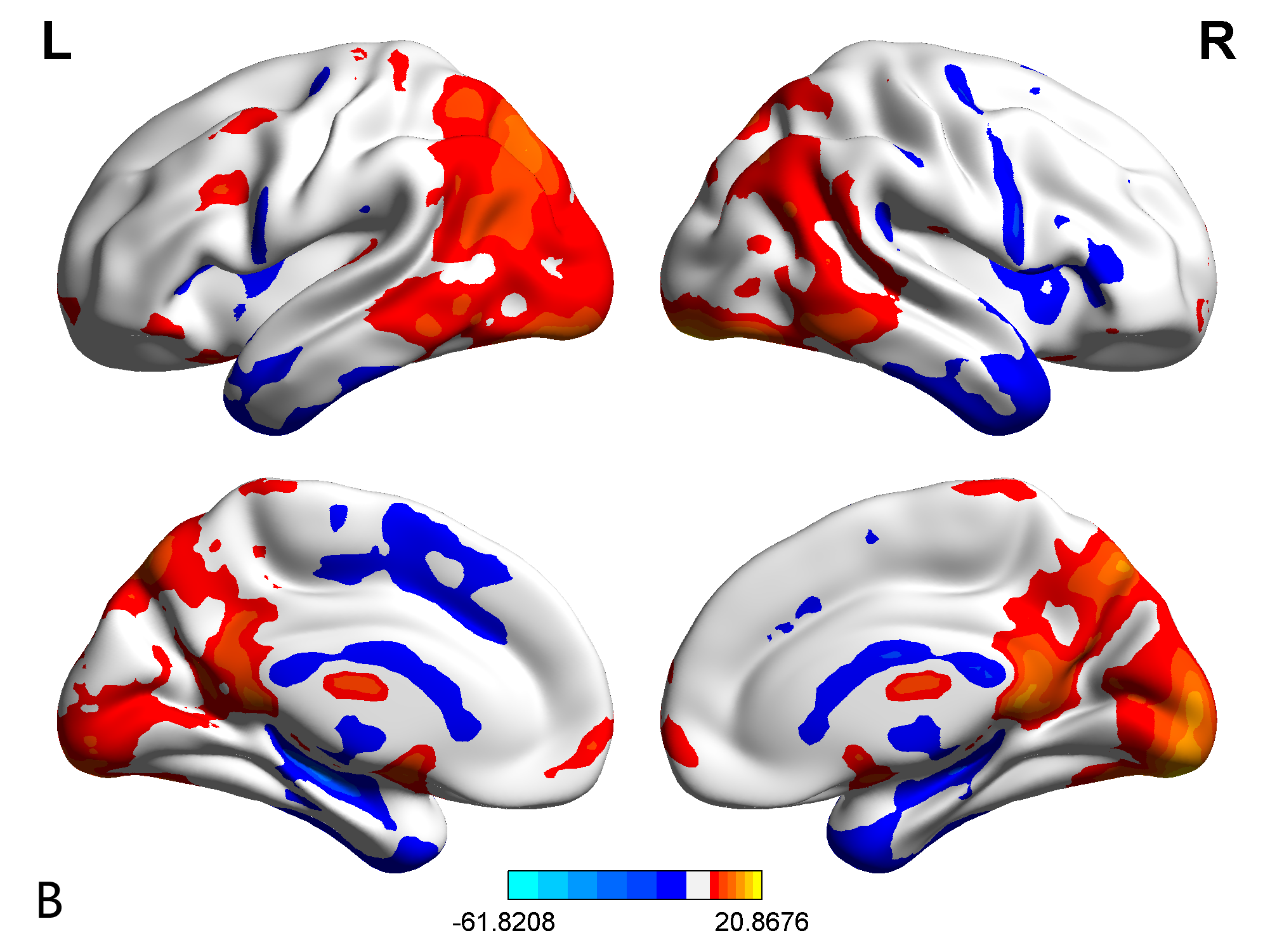

Supplement: Figure S1 — The mean degree centrality (DC) maps of two groups: healthy control (A) and subclinical depression (B). [file DataSheet_1.zip › Suppl Mat 10.3389fnhum.2016.00617/Figure S1B.tif]

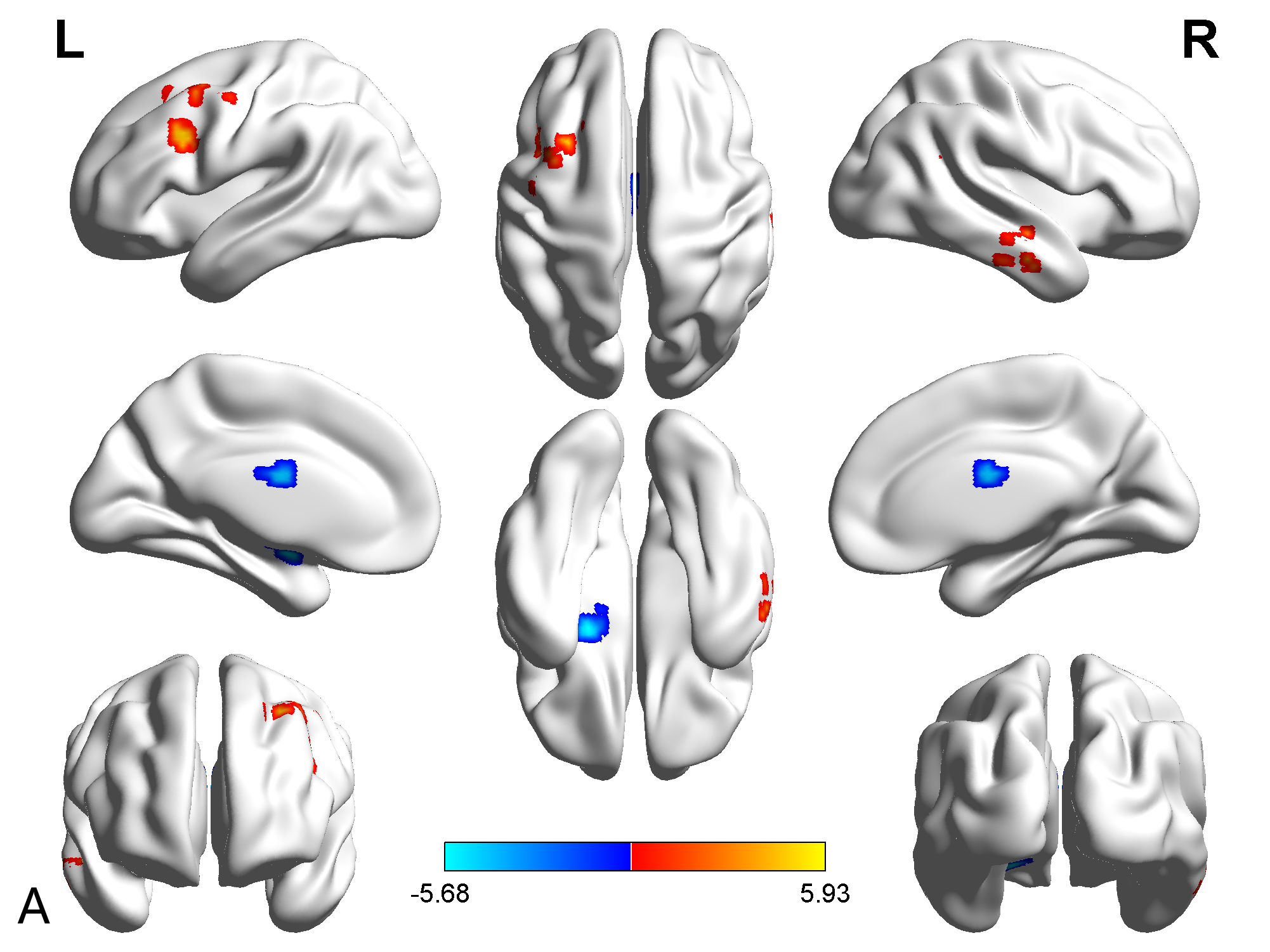

Supplement: Figure S1 — The mean degree centrality (DC) maps of two groups: healthy control (A) and subclinical depression (B). [file DataSheet_1.zip › Suppl Mat 10.3389fnhum.2016.00617/Figure S2A.tif]

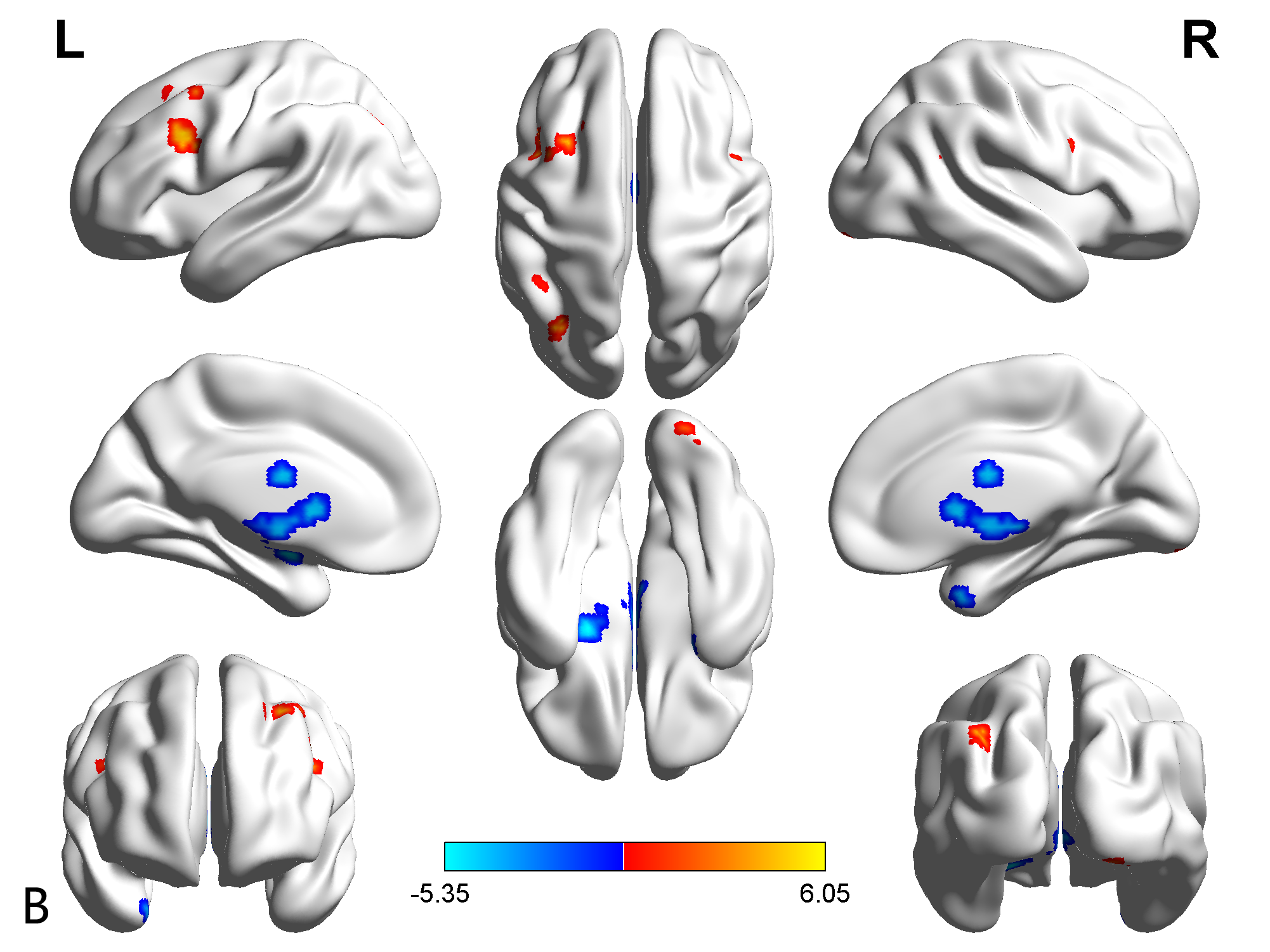

Supplement: Figure S1 — The mean degree centrality (DC) maps of two groups: healthy control (A) and subclinical depression (B). [file DataSheet_1.zip › Suppl Mat 10.3389fnhum.2016.00617/Figure S2B.tif]

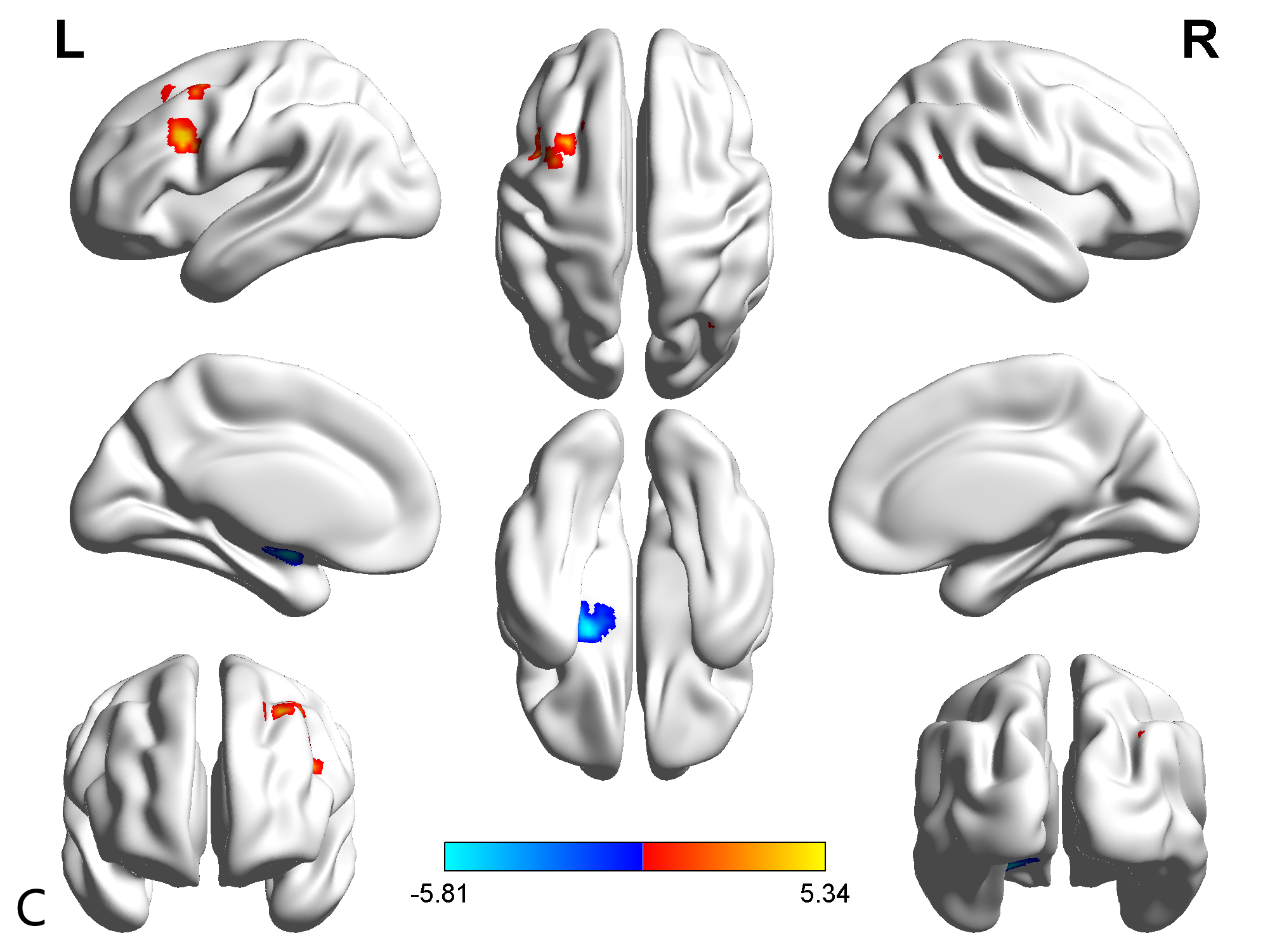

Supplement: Figure S1 — The mean degree centrality (DC) maps of two groups: healthy control (A) and subclinical depression (B). [file DataSheet_1.zip › Suppl Mat 10.3389fnhum.2016.00617/Figure S2C.tif]

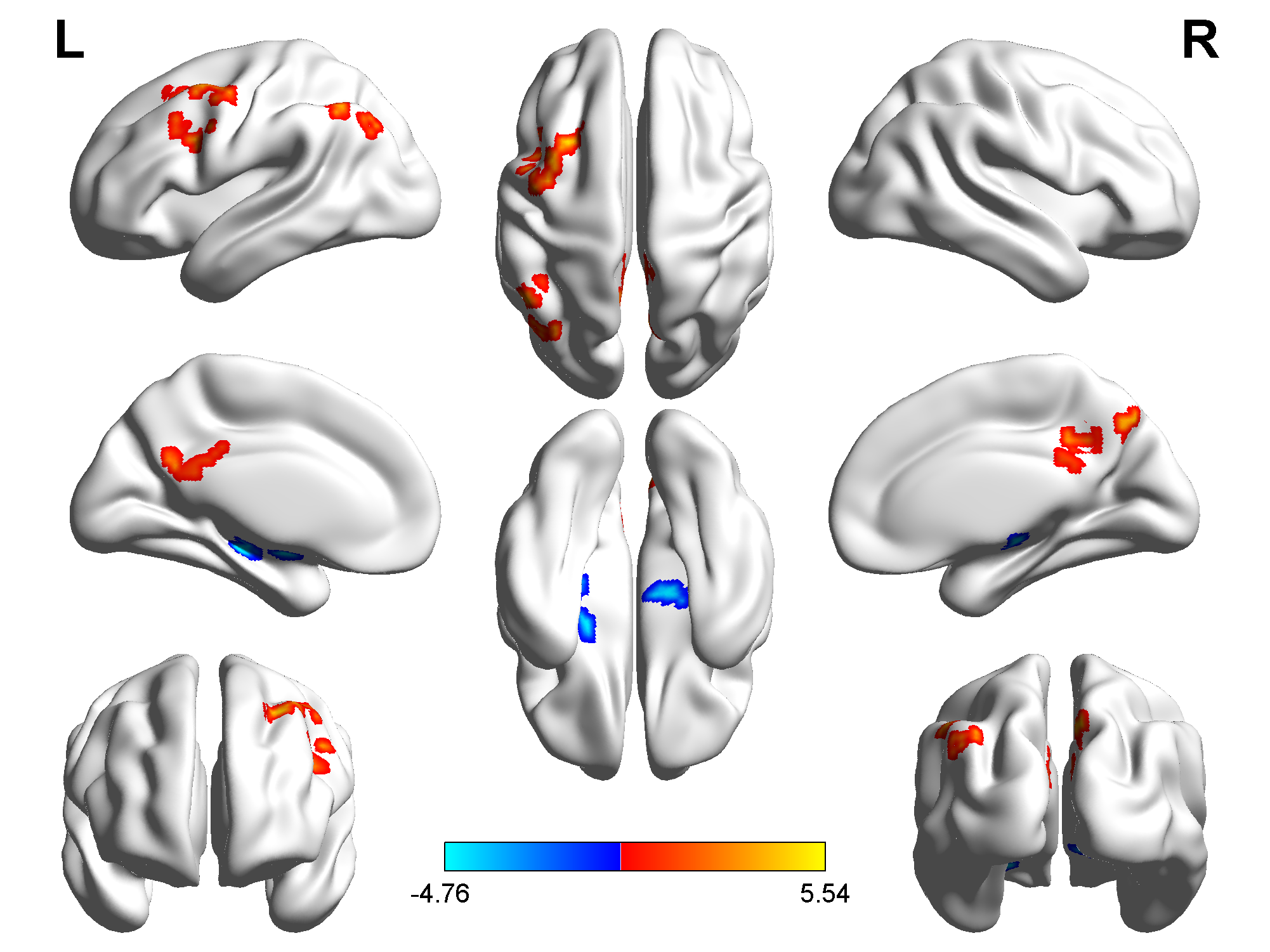

Supplement: Figure S1 — The mean degree centrality (DC) maps of two groups: healthy control (A) and subclinical depression (B). [file DataSheet_1.zip › Suppl Mat 10.3389fnhum.2016.00617/Figure S3.tif]
